# Decoding phenotypic signatures of *Cylas formicarius* Fab. resistance in a global sweetpotato (*Ipomoea batatas* [L.] Lam.) germplasm collection

**DOI:** 10.3389/fpls.2025.1625810

**Published:** 2025-09-26

**Authors:** Alfredo Morales, Peiyong Ma, ZhaoDong Jia, Dania Rodríguez, Iván Javier Pastrana Vargas, Rosa Elena González, Osmany Molina, Alay Jiménez, Yuniel Rodríguez, Lilian Morales, Yoel Beovides, Orelvis Portal, Xiaofeng Bian

**Affiliations:** ^1^ Institute of Food Crops, Jiangsu Academy of Agricultural Sciences (JAAS), Nanjing, China; ^2^ Plant Breeding and Genetic Resources Department, Research Institute of Tropical Roots and Tuber Crops (INIVIT), Villa Clara, Cuba; ^3^ Department of Agronomic Engineering and Rural Development, Faculty of Agricultural Sciences, Universidad de Córdoba, Montería, Colombia; ^4^ Departamento de Biología, Facultad de Ciencias Agropecuarias, Universidad Central “Marta Abreu” de Las Villas, Santa Clara, Cuba; ^5^ Centro de Investigaciones Agropecuarias, Facultad de Ciencias Agropecuarias, Universidad Central “Marta Abreu” de Las Villas, Santa Clara, Cuba

**Keywords:** characterization, germplasm collection, ipomoea batatas, resistance, tuberization depth, weevil

## Abstract

**Introduction:**

Sweetpotato (*Ipomoea batatas* [L.] Lam.) is a critical global food crop that suffers devastating yield losses from the sweetpotato weevil (*Cylas formicarius*), especially in tropical regions where chemical control is often impractical. Breeding for stable resistance has been hindered by an insufficient characterization of reliable phenotypic markers across diverse genetic backgrounds.

**Methods:**

We evaluated 731 accessions from Cuba’s national sweetpotato collection, enriched with global varieties, to identify morphological traits associated with natural resistance to *C. formicarius*. Resistance and susceptibility were assessed through combined field and laboratory bioassays.

**Results:**

Only 6.5% of the accessions demonstrated resistance (<10% infestation), while 80% were highly susceptible. Weak to moderate correlations linked resistance to smoother root surfaces (r = 0.31) and lighter flesh pigmentation (r = -0.38). The strongest correlation was observed with deeper tuberization (r = -0.72). Six Cuban genotypes combined agronomic viability (yield >10 t ha⁻¹) with resistance. Five of these employed deep tuberization as a physical escape mechanism, while one genotype, INIVIT B-25, exhibited shallow tuberization (mean depth 4.53 cm) yet maintained resistance, suggesting a biochemical defense strategy. Under controlled infestation, INIVIT B-2022 demonstrated the strongest antibiosis effect, suppressing adult emergence to just two individuals.

**Discussion:**

Our study decodes key phenotypic signatures of weevil resistance, providing immediately actionable morphological traits for use in Caribbean breeding programs. The discovery of a resistant genotype with shallow roots indicates the presence of a non-escape, potentially biochemical resistance mechanism. This highlights the critical need for subsequent molecular studies to uncover the complementary genetic and biochemical bases of these defenses.

## 1 Introduction

Sweetpotato (*Ipomoea batatas* [L.] Lam.) ranks among the world’s most important food crops for human consumption. Currently, it holds 12th place in global food crop production, with an annual average of 93.5 million tons, making it the 3rd most produced root and tuber crop (after potato and cassava) (FAOSTAT, 2023). Beyond production volume, its nutritional value (particularly vitamin A content among biofortified crops) surpasses most cereals (CIP, 2024). Its short growth cycle, resilience to extreme weather, vegetative propagation, and drought tolerance position it as a key crop for food security in vulnerable tropical regions (Morales et al., 2025).

*Ipomoea batatas* originated through a complex polyploidization process in its wild lineage. Evidence suggests that the first key event was an allopolyploidization occurring approximately 800,000 years ago. Subsequently, the cultivated species emerged via a second hybridization event (4x × 2x), followed by complete genome duplication around 500,000 years ago (Yang et al., 2017; Muñoz-Rodríguez et al., 2018). While human domestication occurred much later in the Neotropical region spanning from southern Mexico to South America archaeological records indicate its cultivation in present day Peru approximately 8,080 years (± 170 BC) (Engel, 1970; Austin, 1988).

*Cylas formicarius* is regarded as the most devastating pest of sweetpotato worldwide (Chalfant et al., 1990). Field studies demonstrate 60-100% yield loss in unprotected plots, with larval tunneling reducing both root quality and marketable yield (Cisneros and Alcázar, 2001). Secondary effects include terpenoid-induced bitterness (Data et al., 1996) and mycotoxin contamination from opportunistic fungi (Stathers et al., 2003). In Cuba, weevil damage typically reduces farmers’ income by 40-100%, with complete crop loss occurring in severe infestations (Cisneros and Alcázar, 2001).

*Cylas formicarius* represents a biogeographical exception within its genus, while all other *Cylas* species are native to Africa, *C. formicarius* originated in Asia (India) (Wolfe, 1991). Phylogenetic studies suggest that its lineages diverged in Asia and the Pacific ~6–13 million years ago, long before the introduction of sweetpotato to these regions (Brookes et al., 2019). Thus, it is likely that *C. formicarius* initially relied on other *Ipomoea* species as native hosts before adapting to sweetpotato (Wolfe, 1991; Brookes et al., 2019).

The earliest potential contact between cultivated sweetpotato and *Cylas formicarius* may have occurred during the Polynesian expansion (~1000 AD), though direct evidence is lacking (Roullier et al., 2013). The weevil’s definitive global dispersal coincided with Portuguese and Spanish trade routes in the 16th century (~1520s), when sweetpotato was introduced to Africa and Asia (Brookes et al., 2019). Thus, their intensive coevolution likely spans only 500–1000 years.

Cuba is presumed to have been the epicenter of the weevil’s distribution in the Caribbean. In 1875, Chinese immigrants arrived on the island to work in sugarcane plantations and may have introduced infested sweetpotatoes from China (Amargos, 1935). Therefore, the interaction between sweetpotato and *C. formicarius* in the Caribbean region dates back only ~150 years.

In 1993, the International Potato Center (CIP) and the Research Institute of Tropical Roots and Tuber Crops (INIVIT) launched a collaborative eight-year research initiative to combat the sweetpotato weevil. This effort led to the development of a highly effective integrated pest management (IPM) program, which achieved significant environmental and health benefits. The IPM strategy reduced weevil damage from 50% to less than 5% and was subsequently adopted across all sweetpotato-growing regions of Cuba (Cisneros and Alcázar, 2001). However, in recent years, pest resurgence has occurred due to limitations in agricultural extension services for IPM. In this context, the development of resistant varieties emerges as a sustainable and economically viable alternative.

Previous studies have identified sweetpotato genotypes with lower infestation rates, attributed to morphological traits such as elongated roots, dispersed tuber arrangement (Cockerham and Deen, 1947), thick cortex, and slender stems (Pole, 1988). However, these traits did not consistently confer resistance under field conditions (Magenya and Smit, 1992; Stathers et al., 2003). Biochemical mechanisms have also been linked to resistance, including the presence of caffeic and coumaric acids, triterpenoids and latex rich in hydroxycinnamic acid esters (Snook et al., 1994; Stevenson et al., 2009), which reduce feeding and oviposition by the weevil (Data et al., 1996).

Although occasional reports have documented high levels of experimental resistance, these proved unstable (Waddill and Conover, 1978; Jones et al., 1980; Talekar, 1987). Key factors contributing to this inconsistency include: non-standardized evaluation methods, genetic complexity of sweetpotato (hexaploidy), multifactorial nature of resistance, and influence of environmental factors (Marti et al., 1991).

The lack of stable resistance sources in *I. batatas* may be explained by its brief co-evolutionary history with *C. formicarius* (~150 years in the Caribbean). Nevertheless, resistance appears to involve multiple mechanisms, including antibiosis, antixenosis (non-preference), and tolerance (Kogan and Ortman, 1978), and is polygenic, expressed as a continuous gradient (Rolston et al., 1979). In Cuba, studies suggest that some varieties exhibit resistance linked to escape mechanisms, such as early maturity and deep tuberization (Cisneros and Alcázar, 2001).

The use of sweetpotato genotypes resistant to the weevil is the most effective control strategy. Unfortunately, no such genotypes currently exist. Despite over 50 years of breeding efforts at INIVIT, no resistant varieties have been developed, those not selected by weevil females for oviposition (non-preference mechanism) or in which larvae fail to develop normally (antibiosis) in stems or storage roots.

Genetic variability is the fundamental basis for the success of any breeding program. Determining the level of variation within collected species is invaluable for both plant breeding and species conservation (Lin et al., 2007), as well as for assessing variability in important traits such as nutrient content and tolerance to biotic and abiotic stresses.

At INIVIT in Cuba, the National Germplasm Collection of Sweetpotato is maintained, comprising 731 accessions: 387 natives, 146 foreign (from China, Japan, Vietnam, United States, various Caribbean islands, Panama, Nicaragua, Colombia, Peru, Brazil, Spain, and Nigeria), and 198 improved varieties. This collection has become the most important in Central America and the Caribbean due to its genetic diversity (Morales et al., 2023, 2024).

This study addresses three critical gaps in sweetpotato weevil resistance research: providing the first comprehensive screening of Cuba’s national germplasm collection (731 accessions), overcoming sample size limitations of prior studies (<200 accessions); simultaneously evaluating field performance and laboratory resistance mechanisms; and identifying genotypes combining agronomic value with resistance traits specifically adapted to Caribbean conditions. While previous research on this collection has characterized yield, nutrition, and climate resilience, the absence of systematic *C. formicarius* evaluation has limited its breeding utility. Our phenotypic approach offers immediately applicable solutions for resource-constrained regions, though we acknowledge future molecular studies will be needed to characterize the biochemical basis of resistance in selected genotypes. Given sweetpotato’s vital role in global food security and the Caribbean’s acute vulnerability to weevil-related losses, this work provides both practical tools for farmers and a foundation for deeper genetic investigation.

## 2 Materials and methods

### 2.1 Study area

The study was conducted at the Research Institute of Tropical Roots and Tuber Crops (INIVIT) in Santo Domingo, Villa Clara, Cuba (22°35´00´´N, 80°14’18´´W; 50 m above sea level), during 2021 and 2024. The site has a calcareous brown soil (Hernández et al., 2015). Meteorological data were recorded at the automatic meteorological station belonging to the institute (national meteorological network code: 78326, data: http://www.insmet.cu).

### 2.2 Basic unit of characterization

A total of 731 sweetpotato accessions were characterized, of which 387 were native, 146 foreign, and 198 were improved (**Table 1**).

**Table 1:** Origin and number of sweetpotato accessions in the Cuban germplasm collection.

Origin of accessions		Number
Natives		387
Improved		198
Non-native germplasm	China	21
	Japan	2
	Vietnam	5
	Mexico	3
	Panama	2
	Nicaragua	5
	EEUU	5
	Peru	28
	Brazil	2
	Argentina	2
	Guadalupe	2
	Jamaica	2
	Martinique	2
	Saint Lucia	1
	Haiti	5
	Spain	1
	Nigeria	50
	Angola	2
	Georgia	4
	Russia	2

### 2.3 Characteristics of the experimental area and agronomic management

**Figure 1:**
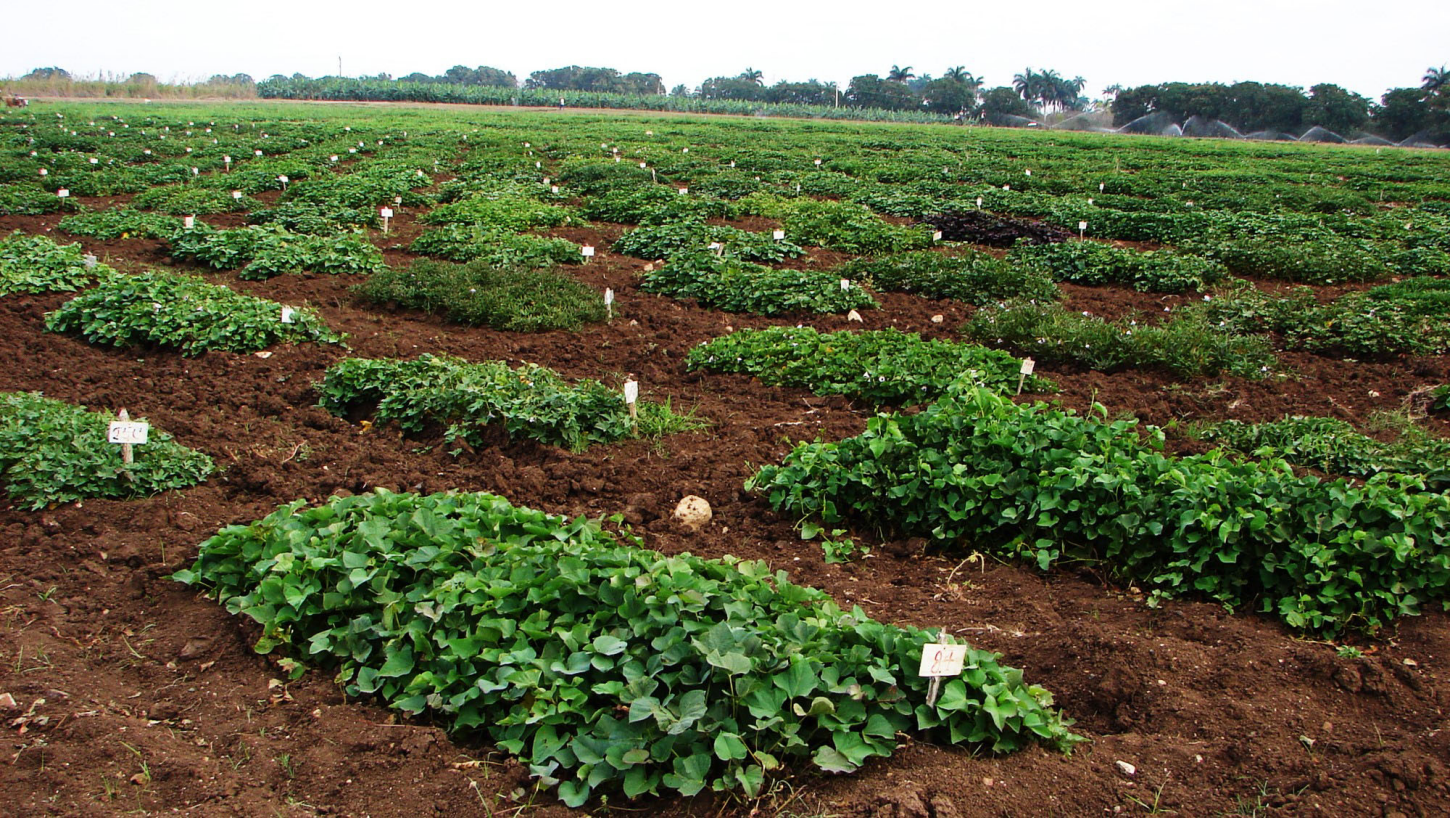
Plots of the sweetpotato collection in the experimental area.

Planting was conducted on October 10, 2021, establishing for each sweetpotato genotype to be characterized, a plot of two rows two meters long (**Figure 1**), for an effective area of 3.6 m^2^, using a planting distance of 0.9 x 0.30 m, with 14 total plants per plot, and a distance between plots of two meters (on both sides). The type of irrigation used was sprinkler, with a weekly frequency, at a net partial norm of 250 m^3^ ha^-1^. No chemical or organic fertilization, nor chemical or biological pesticides were applied. The agronomic management used was that recommended in the Technical Instructions for this crop, proposed by INIVIT (2012).

### 2.4 Variables assessed

A morphological characterization was performed based on 8 qualitative (nominal and ordinal) varietal descriptors (**Table 2**). Additionally, sweetpotato genotypes were characterized using morphometric traits with 16 continuous quantitative variables (**Table 3**), along with an agronomic evaluation of resistance/susceptibility to *C. formicarius*.

**Table 2:** Morphological descriptors used for characterization of the 731 sweetpotato accessions.

Descriptor
Skin color of tuberous roots
Flesh color of tuberous roots
Defects in the skin surface of tuberous roots
Stem color
Color of the insertion point of the petiole and stem
Color of young leaf
Flowering

Descriptors adapted from CIP, AVRDC, IBPGR (1991).

**Table 3:** Morphometric descriptors used in the characterization of the 731 sweetpotato accessions.

Variables	Code	Formula	No.
Flesh luminosity	L*f	–	
Flesh a* coordinate	a*f	–	
Flesh b* coordinate	b*f	–	
Skin luminosity	L*k	–	
Skin a* coordinate	a*k	–	
Skin b* coordinate	b*k	–	
Root circularity	Croot	$\frac{4\pi \times \left(A\right)}{P^{2}}$	(1)
Root aspect ratio	ARroot	$\frac{Ema}{Emi}$	(2)
Root roundness	Rroot	$\frac{4\times A}{\pi \times md^{2}}$	(3)
Root solidity	Sroot	$\frac{A}{Ca}$	(4)
Leaf area	A	–	
Leaf perimeter	P	–	
Leaf circularity	C	$\frac{4\pi \times \left(A\right)}{P^{2}}$	(5)
Leaf aspect ratio	AR	$\frac{Ema}{Emi}$	(6)
Leaf roundness	R	$\frac{4\times A}{\pi \times md^{2}}$	(7)
Leaf solidity	S	$\frac{A}{Ca}$	(8)

A, area; P, perimeter; md, maximum diameter; Ca, convex area; Ema, major axis; Emi, minor axis.

All morphological traits were described at 90 days after planting (DAP). Quantitative descriptors were recorded from the means obtained from 10 plants per accession, while qualitative descriptors were derived from the average expression of the trait observed in the section located at the center of the main stem.

Of the 16 morphometric variables used, 10 were focused on the shape, dimensions, and color of storage roots, and six on the shape and dimensions of leaves. Samples were individually photographed using a Canon EOS 600D camera (Fukushima Canon Inc., Fukushima, Japan). For root color quantification: washed roots were gently patted dry for skin color measurements to ensure surface consistency, while flesh color was assessed immediately after making fresh transverse cuts to prevent oxidation effects. Three measurement points were recorded for both skin and flesh on each of three representative roots per accession.

Measurements were conducted under controlled environmental conditions: temperature (20 ± 2°C), relative humidity (75 ± 5%), and illumination (500 lx). For shape quantification of roots and leaves, we used the professional digital image analysis software ImageJ version 1.46 (NIH, USA), programmed in Java. The color space employed was the CIE 1976 L*a*b* system from the International Commission on Illumination, where: L* = lightness, a* = red/green coordinates (+a indicates red, -a indicates green), and b* = yellow/blue coordinates (+b indicates yellow, -b indicates blue).

In August 2022 (10 months after planting), we conducted an agronomic evaluation of resistance/susceptibility to *C. formicarius*. All roots were harvested by accession and collected individually. The infestation percentage was determined per cultivar:


$$\text{\% infestation}=\;\frac{\text{Weight of infested roots}}{\text{Total weight of roots }}*100$$

Scale:

Resistant = 0-10% infestation
Tolerant = 11-30% infestation
Susceptible = > 31% infestation

### 2.5 Field evaluation of resistance/susceptibility to *C. formicarius*

Following the identification of putative resistant accessions from the germplasm collection, experimental trials were conducted with these selections. Plantings were established in September 2022 (dry season) across two spatially separated fields (1,500 m apart) within INIVIT’s experimental station. The trials employed Talekar’s (1982) methodology, featuring. Two border rows (0.90 m wide, spaced 2.70 m apart) planted two months prior to the test genotypes using the susceptible cultivar ‘CEMSA 78-354’ to establish uniform pest pressure. Two central rows maintained as buffer zones. Following rotary tillage of central rows, test genotypes were planted in two row plots (0.90 m × 5 m x 2 = 9 m²) with three replicates per genotype. This design positioned each genotype equidistant from infested borders, ensuring consistent weevil exposure. No chemical or biological insecticides were applied throughout the trial period.

Harvest occurred at 150 days after planting (DAP), with all storage roots collected and evaluated individually by genotype. Infestation percentages were calculated using standardized protocols consistent with previous experiments. Dual trait selection was performed with independent thresholds: minimum yield of 3 t ha^−1^ and maximum infestation of 10%.

The most promising resistant genotypes identified in the 2022 trials were reevaluated in March 2023 using identical methodology. Final harvest occurred at 150 DAP in August 2023, maintaining consistent evaluation parameters across both trial periods.

### 2.6 Resistance/susceptibility to *C. formicarius* under laboratory conditions

In January, February, and March 2024 (an experiment conducted three consecutive times), the sweetpotato genotypes with the highest reported resistance to the weevil were evaluated, along with a susceptible control (CEMSA 78-354). The experiments were conducted under controlled laboratory conditions to determine the level of infestation and the subsequent emergence of adult weevils.

**Figure 2:**
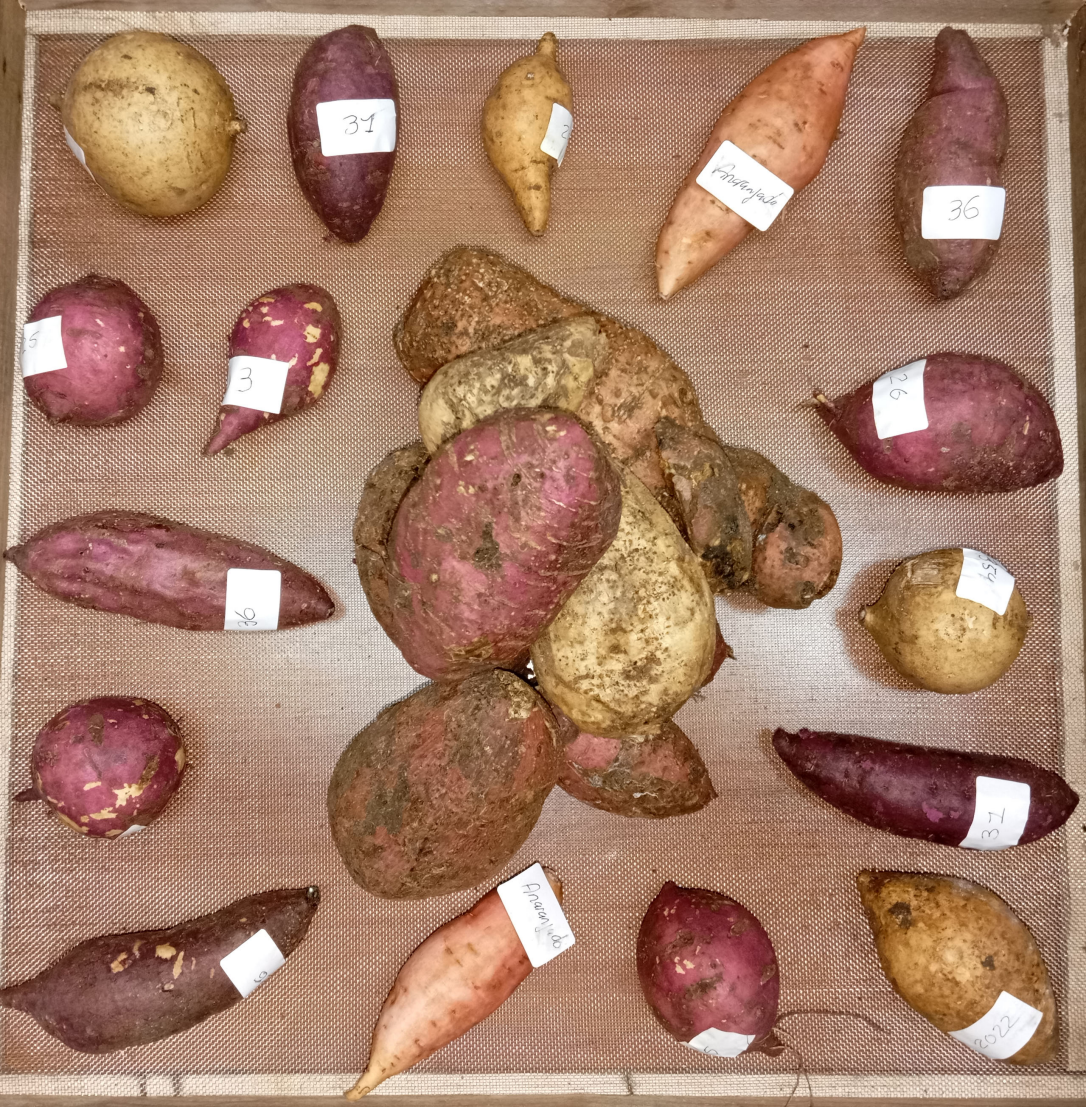
Experimental scheme of controlled infestation with *C formicarius* in sweetpotato genotypes.

The trials were carried out in a square wooden box (50 × 50 × 30 cm) covered with anti-aphid mesh to prevent external contamination while allowing adequate ventilation. Inside the box, sweetpotato roots of each genotype were randomly arranged around a central inoculum source composed of heavily infested sweetpotatoes (previously confirmed to have the presence of *C. formicarius* larvae and adults) (**Figure 2**). This arrangement ensured homogeneous exposure of the genotypes to insect attack.

After one month, the percentage of flesh damage in each sweetpotato was quantified by image analysis using ImageJ version 1.46 software (NIH, USA). To do this, cross sections of the roots were made, and each slice was photographed under standardized conditions. The total flesh area and the area damaged by *C. formicarius* were measured, and the percentage of damage was calculated for each genotype.

After 15 days of initial colonization in the infestation chambers, roots were individually transferred to ventilated Magenta containers (GA-7 vessels, 77×77×97 mm) to monitor adult emergence. This protocol prevented cross genotype contamination. Weevil counts were conducted daily from day 10 to day 14 post-transfer, with a final recording on day 24, enabling quantification of total emerged adults per genotype, temporal emergence patterns, and developmental time from infestation to adult emergence.

### 2.7 Statistical data management and processing

To characterize the accessions while simultaneously considering multiple traits and their interrelationships, we applied interdependence multivariate methods. The analytical procedure involved two main steps: an ordination approach (principal component analysis, PCA) followed by a classification method (cluster analysis).

For continuous quantitative variables, we performed PCA to identify associations between descriptors and determine whether they contributed similarly or oppositely to the observed variation. The eigenvector matrix was interpreted to assess variable contributions. A bar plot was constructed to display the absolute and cumulative proportion of variance (Y-axis) explained by each principal component (X-axis). Correlations between the original variables and selected principal components were calculated using Pla’s (1986) formula. Additionally, variable correlations were projected onto the first two principal axes (PC1 and PC2) to visualize their relationships.

To classify accessions into relatively homogeneous groups based on shared characteristics, we conducted a cluster analysis combined with a heatmap. For multi-state (nominal and ordinal) morphological data, Gower’s (1971) metric distance (dissimilarity) was applied, while Euclidean distance (ED) was used for continuous morphometric variables. An agglomerative hierarchical clustering method (multi-level grouping) was employed, with results presented as a dendrogram.

### 2.8 Data visualization

Analyses were performed in R version 4.0.0 using various packages for statistical processing and data visualization. Graphical representation included bar charts and scatter plots using the ggplot2 package, correlation analysis using networks implemented in the corrr package, complemented by correlation network visualizations using qgraph, chord diagrams to visualize associations between variables using the circlize package, and combined representations of dendrograms and heatmaps generated with ComplexHeatmap. Data were standardized using the scale() function when necessary. All figures were exported in vector format (600 dpi).

## 3 Results

### 3.1 Phenotypic diversity of the germplasm collection and its relationship with resistance/susceptibility to *C. formicarius*

**Figure 3:**
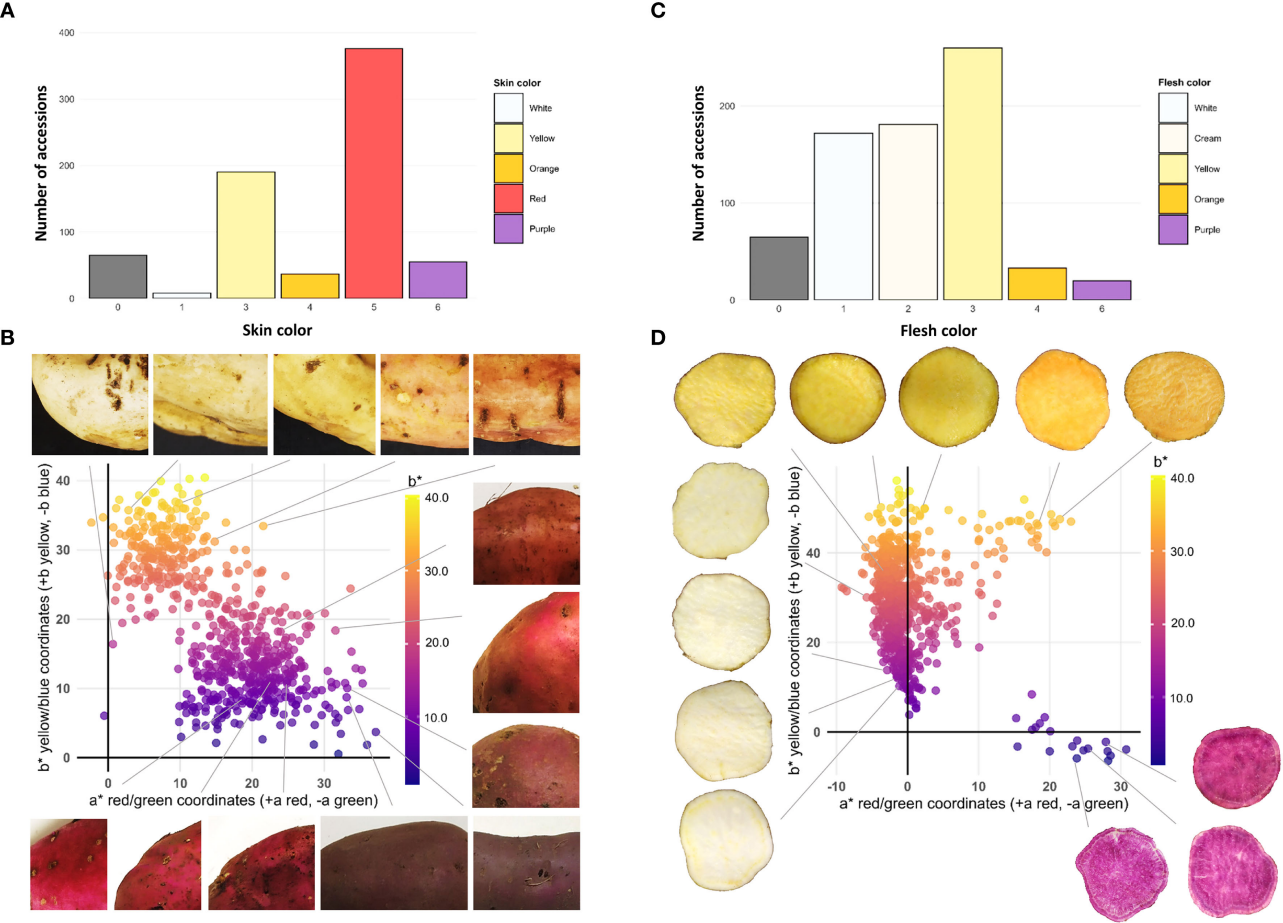
Phenotypic diversity of tuberous root color in the sweetpotato germplasm collection. **(A)** Frequency histogram for skin color; **(B)** Scatter plot for skin color; **(C)** Frequency histogram for flesh color; **(D)** Scatter plot for flesh color.

Out of the 731 accessions, 66 (9%) did not produce tuberous roots. The skin color histogram reveals a clear predominance of red (376 accessions) and yellow (190 accessions) values. The low frequency of white (8 accessions) and purple (55 accessions) skins suggests that these traits are minority features in the collection (**Figure 3A**). The scatter plot generated from the CIELab color space coordinates a* (green-red) and b* (blue-yellow) for the skin of the analyzed accessions shows a heterogeneous distribution, indicating broad variability in the chromatic characteristics of the evaluated samples. The points in the graph exhibit wide dispersion, with a* values ranging approximately from –5 to 35 and b* values from 0 to 40. This variability suggests that the accessions display a diverse range of skin tones, from whitish hues (negative a* values) to reddish tones (positive a* values), as well as from purplish (negative b* values) to yellowish (high b* values) (**Figure 3B**).

The flesh color distribution shows a notable frequency of yellow (260 accessions), cream (181 accessions), and white (172 accessions), followed by orange (33 accessions) and purple (20 accessions) in smaller proportions (**Figure 3C**). The CIELab color space scatter plot for flesh color reveals a concentration of accessions within the a* range of –5 to 5 and the b* range of 0 to 40, suggesting that most samples exhibit intermediate tones with a slight tendency toward whitish and yellowish colors (**Figure 3D**).

**Figure 4:**
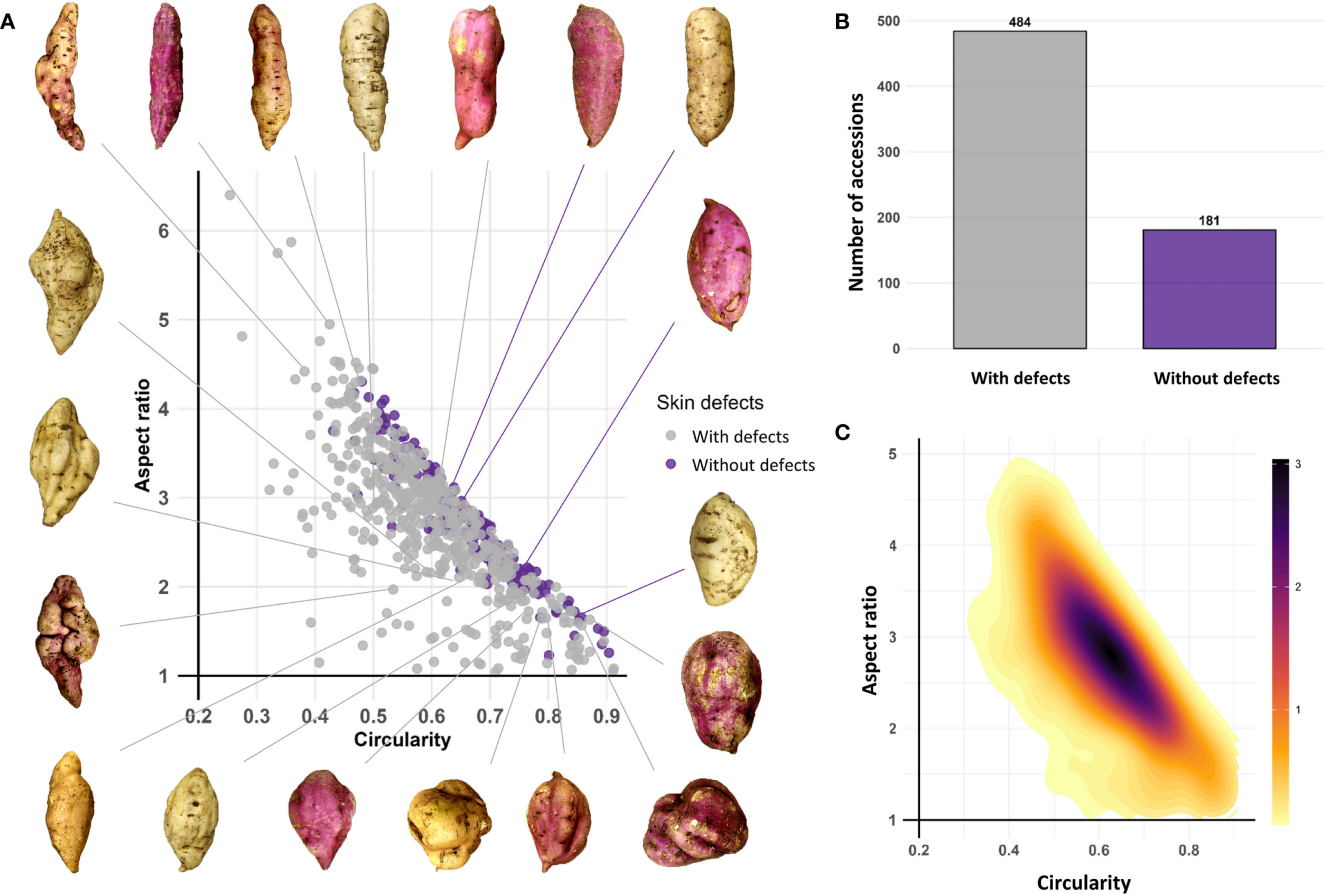
Phenotypic diversity of tuberous root shape in the sweetpotato germplasm collection. **(A)** Circularity **(C)** vs. Aspect Ratio (AR) distribution plot; **(B)** Bar plot for roots with and without defects; **(C)** 2D density plot for Circularity **(C)** vs. Aspect Ratio (AR).

Among the 665 accessions that produced tuberous roots, 484 (72.78%) exhibited skin surface defects (longitudinal fissures and horizontal constrictions), while 181 (27.22%) were defect-free. The Circularity (C) vs. Aspect Ratio (AR) distribution plot reveals significant morphological patterns in the analyzed sweetpotato germplasm. The density distribution shows a predominant concentration in high circularity values (0.5–0.7) and aspect ratio values (2.5–3.5). Low-density areas in regions of low circularity (< 0.4) and high aspect ratio (> 4) indicate a lower frequency of roots with pronounced elongated shapes. Defect-free roots were scattered without a defined pattern (**Figures 4A, B**).

The 2D density plot uses a color gradient to highlight that the highest-density zones (dark tones) correspond to intermediate to high values of both traits, while peripheral areas (yellow tones) represent less common morphologies (**Figure 4C**).

**Figure 5:**
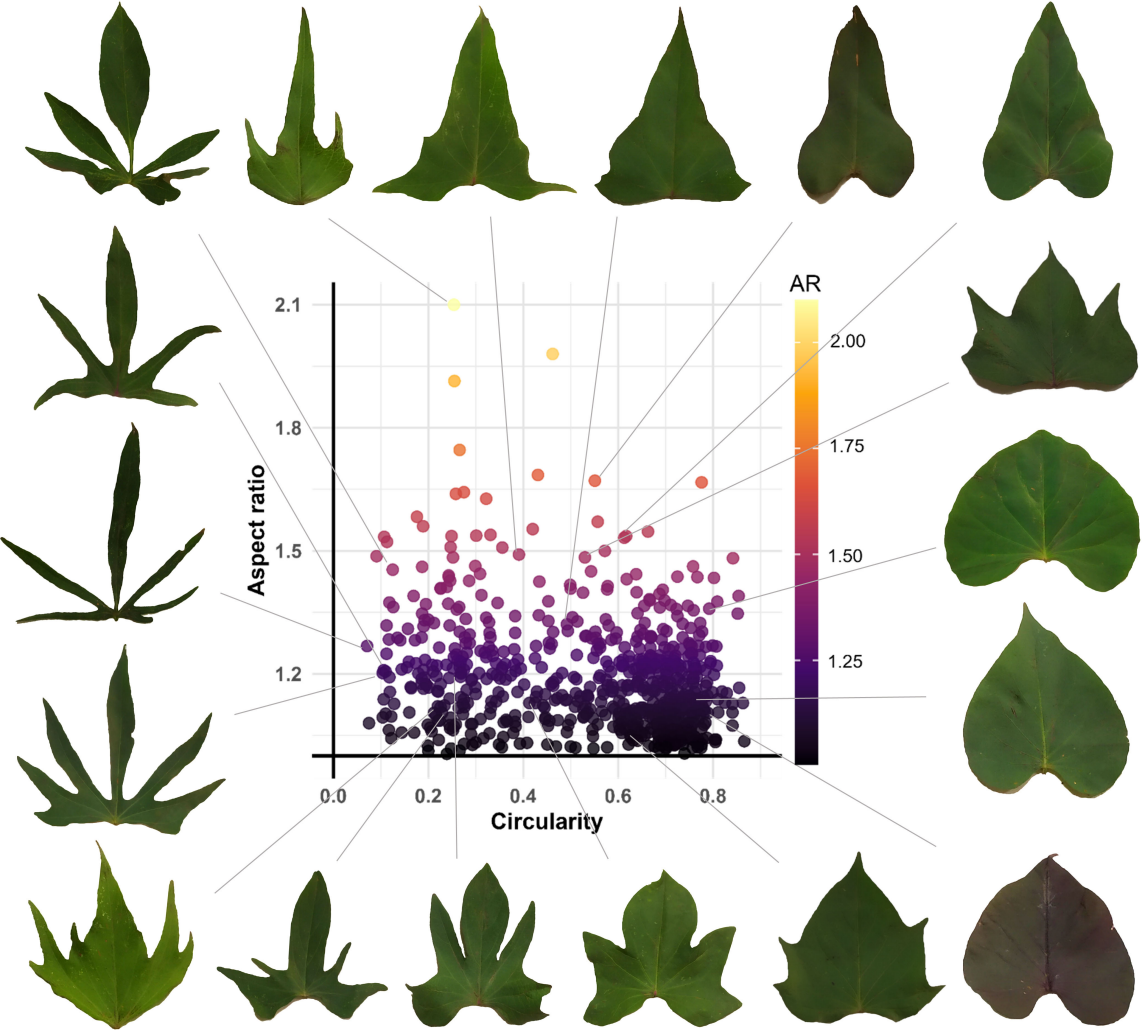
Phenotypic diversity of leaf shape in the sweetpotato germplasm collection.

The analysis of the scatter plot between Leaf Circularity and Aspect Ratio in the sweetpotato germplasm revealed no positive correlation between these variables. However, the observed data dispersion indicates significant variability in leaf morphology among accessions, suggesting differences between these parameters and leaf contour complexity. The density distribution shows a predominant concentration in high circularity values (0.6–0.8) and aspect ratio values (1–1.3), implying that most accessions have relatively compact leaves with well-defined contours. Several outlier points were identified, corresponding to accessions with: highly lobed leaves (low circularity:< 0.2) or exceptionally compact leaves (high circularity: > 0.7) (**Figure 5**).

**Figure 6:**
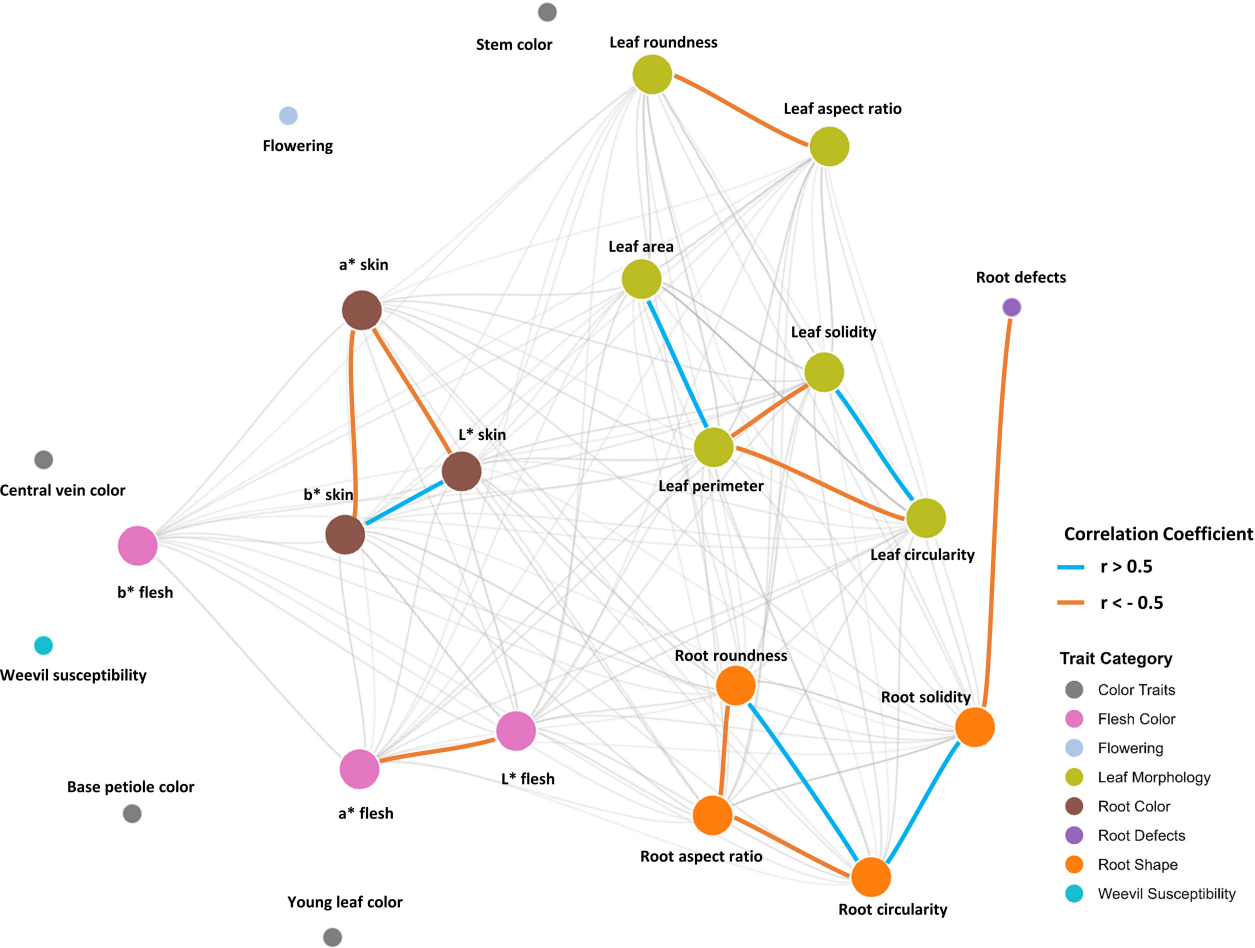
Correlation network graph of the studied variables.

The network graph obtained reveals interesting patterns of interrelationship among phenotypic traits in the sweetpotato germplasm. The network structure displays clear modularity, with traits grouping according to their nature, showing four main modules: tuberous root skin color traits, tuberous root flesh color traits, leaf morphometric traits, and root morphometric traits. The strongest connections (thicker edges, r ≥ 0.5 and r ≤ -0.5) are observed between shape and color parameters within the same tissue, particularly among the L*, a*, and b* coordinates, suggesting highly coordinated pigmentation patterns. Notably, root morphometric traits (circularity, aspect ratio, roundness, and solidity) exhibit high interconnectivity, possibly indicating a shared genetic basis or strong ontogenetic integration during storage organ development. However, the variable of susceptibility to the weevil did not show significant correlations with any of the other morphological or morphometric characters analyzed. The network also highlights a significant bridge between tuberous root defects and root solidity (**Figure 6**).

**Figure 7:**
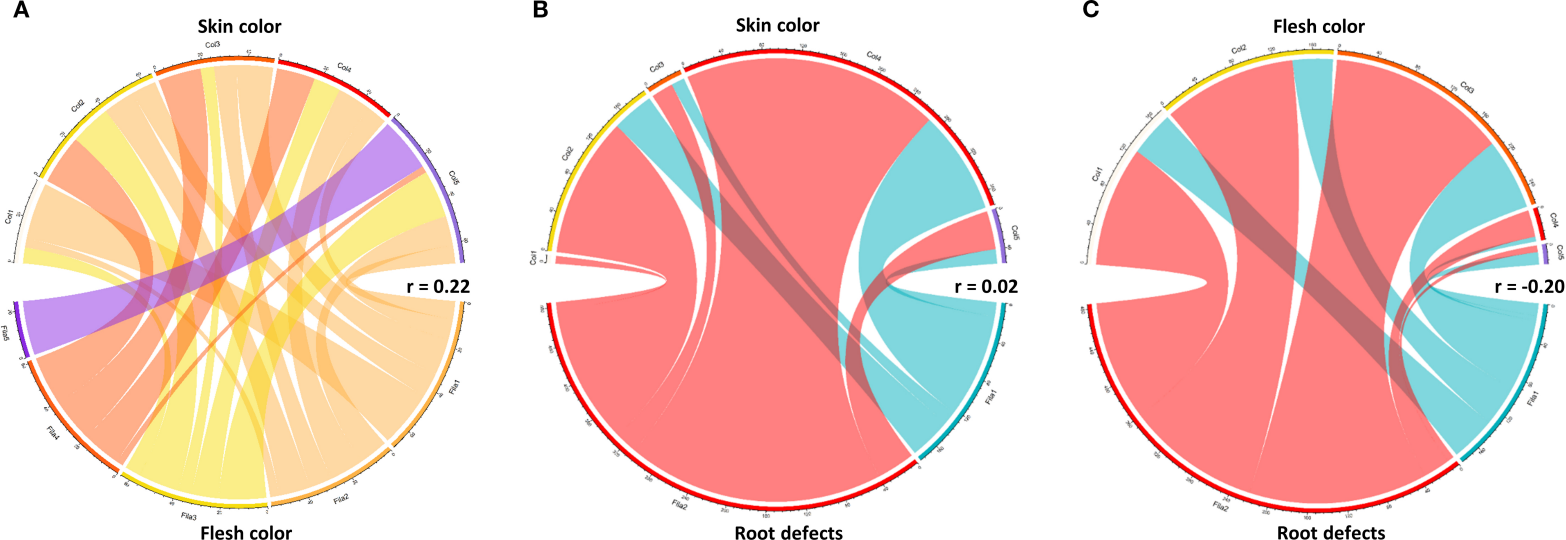
Relationships between coloration and defects in sweetpotato tuberous roots. **(A)** Skin color vs. flesh color; **(B)** Skin color vs. defect presence; **(C)** Flesh color vs. root defect.

The results demonstrate a clear association between skin color and flesh color in sweetpotato tuberous roots. The most frequent combination was red skin with yellow flesh (160 cases), followed by red skin with cream flesh (105 cases), suggesting that genotypes with red skin tend to exhibit yellow or cream flesh. In contrast, purple skin showed exclusivity with purple flesh (20 cases) (**Figure 7A**).

A higher incidence of defects was observed in roots with red skin (268 defectives vs. 108 defect-free) and yellow skin (146 defectives vs. 44 defect-free). Conversely, roots with white skin showed the lowest defect frequency (8 cases) (**Figure 7B**). Roots with yellow flesh had the highest defect count (186 cases), while those with purple flesh showed the lowest incidence (7 cases). Notably, orange flesh though less frequent exhibited a substantial proportion of defects (28 cases), indicating a non-linear relationship between color and defect susceptibility (**Figure 7C**).

**Figure 8:**
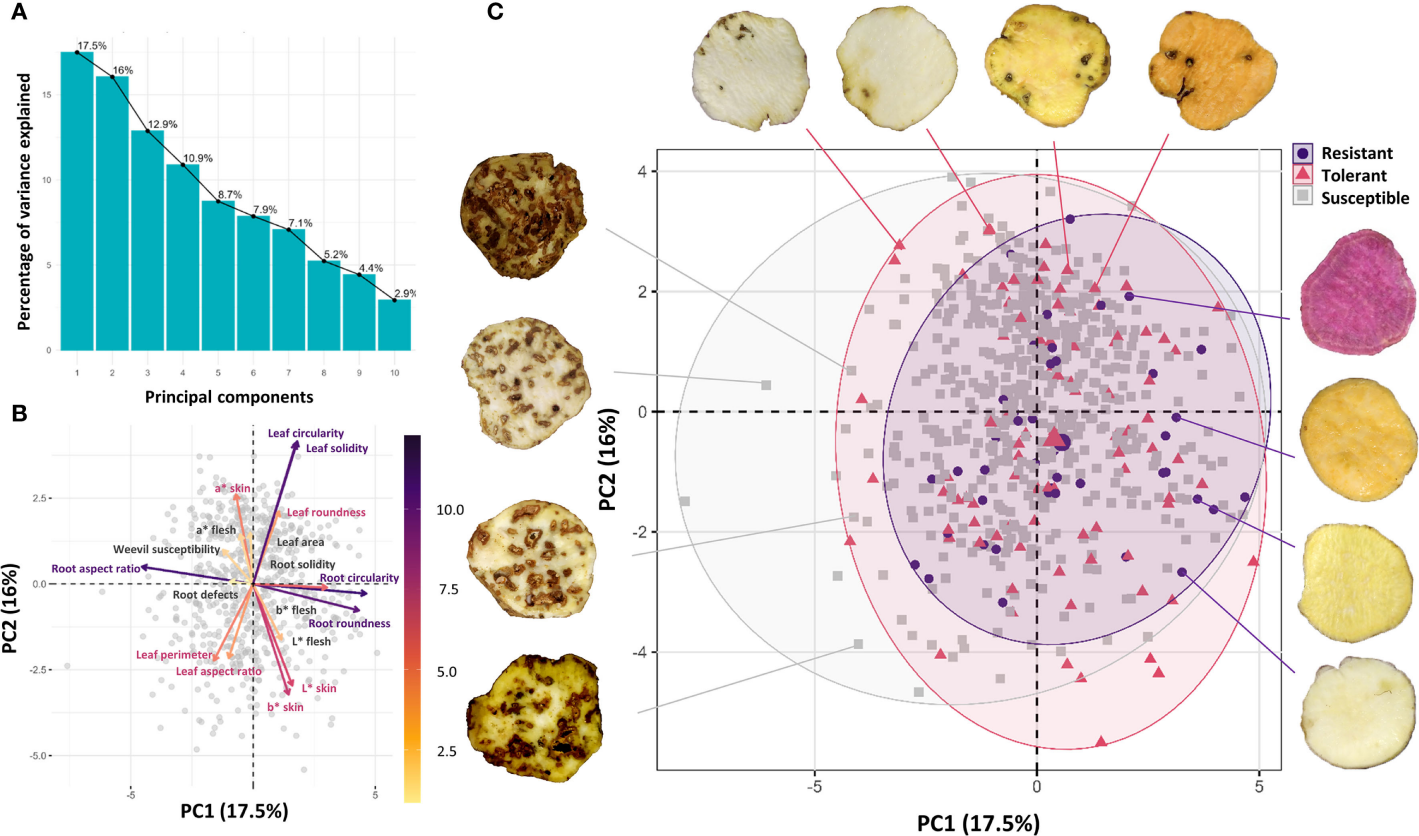
Principal Component Analysis (PCA) of variables in the sweetpotato collection. **(A)** Proportion of variance explained by each component; **(B)** Variables projected in the factorial plane (Component 1 vs. Component 2); **(C)** Distribution of accessions (susceptible, tolerant, and resistant to C. formicarius) in factorial space.

The variance associated with each principal component differed and decreased sequentially. The first component explained 17.5% of total variance and the second accounted for 16%. Together, the first four components explained 57.3% of the cumulative variance (**Figure 8A**). Leaf morphometric variables (Circularity and Solidity) showed strong positive correlations with each other and were the primary contributors to Component 1 variance. In contrast, tuberous root morphometric variables (Circularity and Roundness) were highly correlated and represented the main variance contributors for Component 2. The weevil susceptibility variable, located near the origin, indicated little to no relationship with either component (**Figure 8B**).

Sweetpotato accessions clustered into three distinct groups in the factorial space based on their response to *C. formicarius*: susceptible (532 accessions, 80%), tolerant (90 accessions, 13.5%), and resistant (43 accessions, 6.5%). The overwhelming predominance of susceptible genotypes reflects the high vulnerability of the evaluated germplasm. While scarce, the resistant accessions represent valuable genetic resources. Notably, these resistant accessions did not form defined clusters in the biplot but were instead dispersed throughout the principal components space. This pattern suggests that resistance is not associated with the evaluated morphological traits (**Figure 8C**).

Of the 43 accessions that showed resistance, 36 are Cuban, 1 from Japan (Ja-pan-1-2016), 2 from China (YAN SHU-1, YUI-BEI-BUINI), 2 from Nigeria (KOKEINO, IITA-TIS-8250), 1 from Mexico (Mexico-20) and 1 from the United States (Excel).

**Figure 9:**
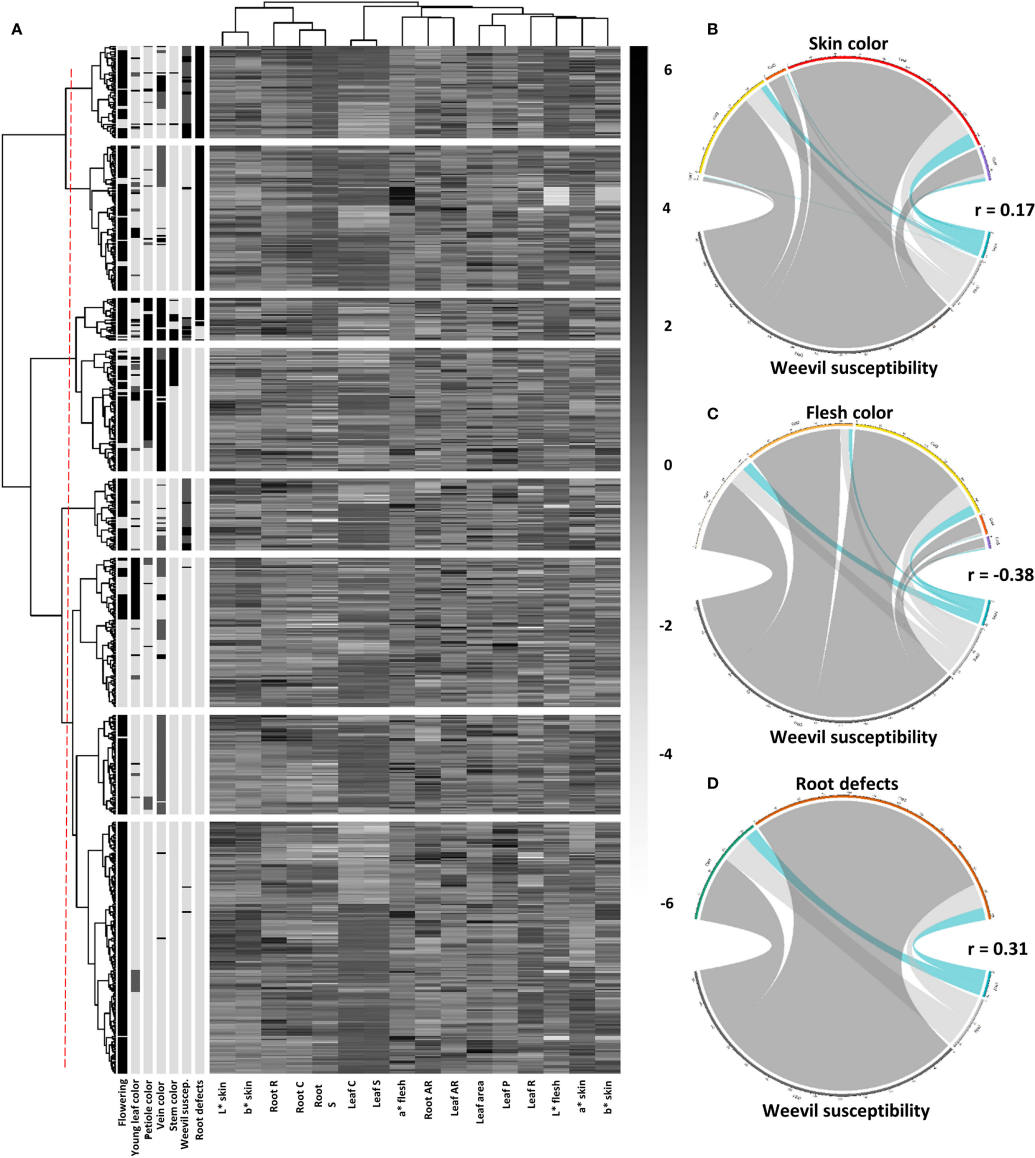
Integrated analysis of sweetpotato phenotypic characteristics and their relationship with resistance to *C. formicarius*. **(A)** Hierarchical clustering and phenotypic profile (heatmap with dendrograms); **(B)** Association between skin color and resistance levels; **(C)** Association between flesh color and resistance levels; **(D)** Association between root defects and resistance levels.

The combined heatmap and dendrogram analysis revealed interesting patterns regarding the susceptibility, tolerance, and resistance of sweetpotato accessions to weevils. Resistant accessions tended to exhibit lighter skin coloration (high L*skin values and low a*skin values) and less pigmented flesh (low b*flesh values). Many of these resistant accessions also showed greater root solidity. In contrast, tolerant accessions occupied an intermediate position in the dendrogram, sharing characteristics with both resistant and susceptible groups. These tolerant accessions typically dis-played moderate skin pigmentation (intermediate a*skin values) and mild surface defects. The susceptible accessions clustered in distinct branches of the dendrogram, often associated with darker skins (high a*skin values) and intensely pigmented flesh (high b*flesh values). Notably, these susceptible accessions frequently presented more pronounced surface defects, which likely facilitates infestation. A particularly relevant finding was the apparent correlation between stem color and weevil susceptibility. Accessions with purple stems tended to be more susceptible. Regarding the relationship between susceptibility to the weevil and the morphometric characteristics of the leaf, the analysis did not show significant associations. The parameters of circularity, aspect ratio and leaf solidity did not show clear patterns that would allow linking the morphology of the leaves with resistance or susceptibility to the insect. (**Figure 9A**).

The data revealed a weak but positive correlation (r=0.17) between skin color and resistance to *C. formicarius*. Red skinned accessions contained the highest number of resistant individuals (25), followed by yellow (11) and purple (6) skins. However, the high proportion of susceptible individuals across all skin colors (particularly in red: 306 susceptible versus 25 resistant) suggests that this morphological trait alone is not a reliable predictor of resistance. White skinned accessions showed the lowest number of resistant individuals (1) (**Figure 9B**).

A moderate negative correlation (r=-0.38) was observed between flesh color and resistance to *C. formicariu*s. White and yellow flesh accessions concentrated the highest number of resistant individuals (17 each), while cream and orange flesh accessions showed greater susceptibility. Particularly striking was the case of purple flesh, which presented only 2 resistant accessions (**Figure 9C**).

A moderate positive correlation (r=0.31) was found between the presence of surface defects (fissures and constrictions) and susceptibility to *C. formicarius*. Among susceptible accessions, 79% exhibited defects, while resistant accessions showed a similar proportion with and without defects (49% versus 51%). This pattern suggests that surface defects may result from insect damage rather than representing an intrinsic trait, though it could also indicate that malformed roots are more vulnerable to attack. The presence of 21 resistant accessions with defects raises the hypothesis that some genotypes may develop tolerance mechanisms, while others exhibit antixenosis resistance (insect avoidance) (**Figure 9D**).

### 3.2 Field evaluation for resistance/susceptibility to *C. formicarius*

**Figure 10:**
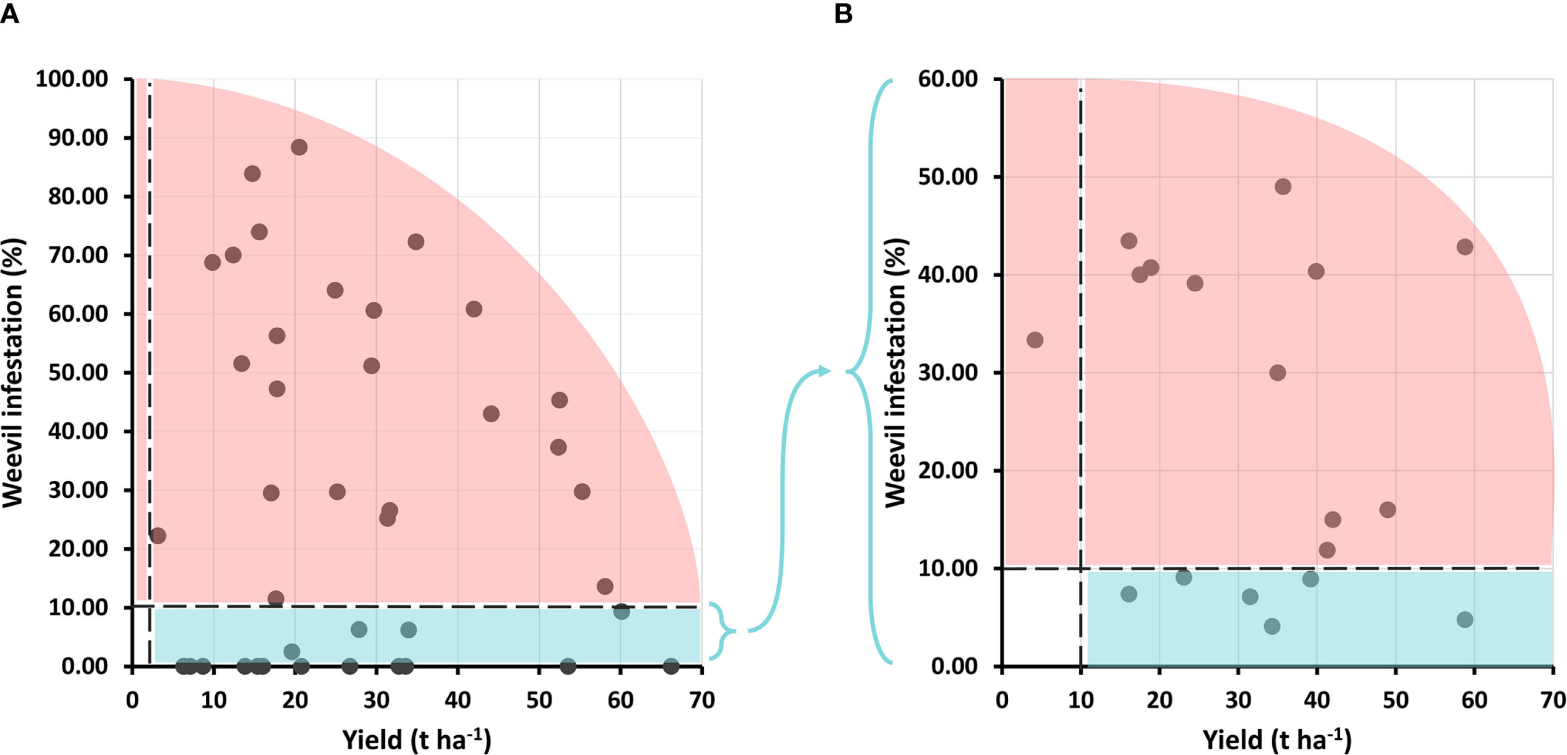
Selection of genotypes based on independent criteria for yield and infestation percentage by *C. formicarius*. **(A)** First evaluation cycle (43 genotypes); **(B)** Second evaluation cycle (18 genotypes).

From the 43 accessions initially identified as resistant in the germplasm collection screening, only 18 genotypes met the dual selection criteria (yield ≥3 t ha^−1^ and infestation ≤10%) in the first experiment (designed to assess resistance). While accessions with outstanding combinations of yield and resistance were identified such as INIVIT B-25 (60.13 t ha^−1^ with 9.31% infestation) most genotypes exhibiting complete resistance (0% infestation) showed moderate yields (<20 t ha^−1^), revealing a clear trade-off between these traits. However, two exceptional genotypes exceeded 50 t ha^−1^ with 0% infestation (**Figure 10A**).

In a second evaluation cycle, only 6 of the 18 tested genotypes met the dual selection criteria, simultaneously achieving yields above 10 t ha^−1^ and infestation below 10%. These were classified into three groups based on performance. High yield and resistance: Highlighted by INIVIT B-25 (58.8 t ha^−1^ with only 4.76% infestation), which emerged as the most promising genotype due to its exceptional productivity and phenotypic stability. Good yield and good resistance: Represented by INIVIT B-2022 (39.2 t ha^−1^, 8.93%), IB Morado-16 (34.3 t ha^−1^, 4.08%), and INIVIT B-90 (31.5 t ha^−1^, 7.11%). Moderate yield but good resistance: comprising Catalina (23.1 t ha^−1^, 9.09%) and V-525 (16.1 t ha^−1^, 7.39%) (**Figure 10B**). All six resistant genotypes are of Cuban origin, five being improved varieties and one traditional (Catalina).

**Figure 11:**
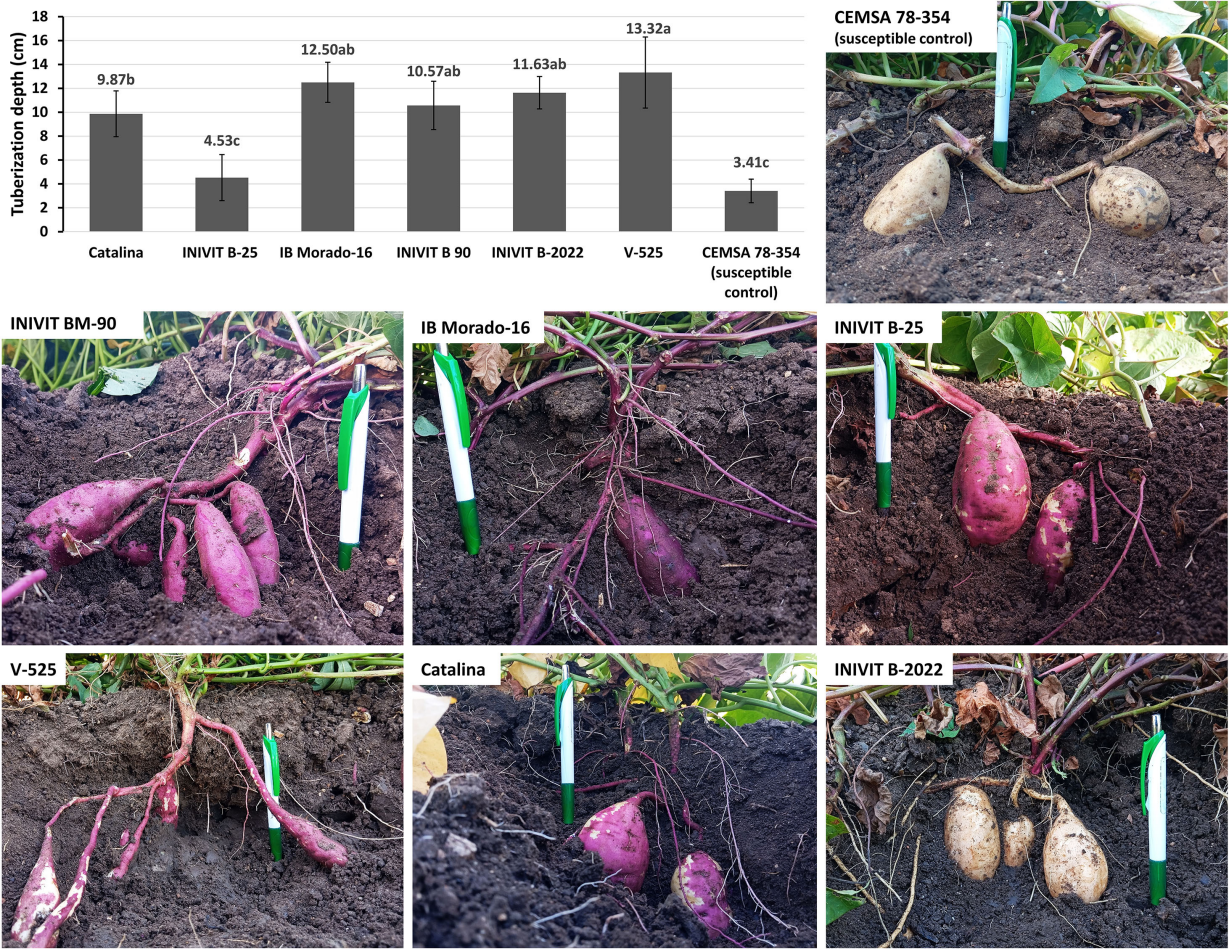
Tuberization depth of the six genotypes with the highest resistance to *C. formicarius* and the susceptible control genotype. Photos taken 80 days after planting.

Highly significant differences (p< 0.05) were found in the tuberization depth among the evaluated sweetpotato genotypes (six resistant and one susceptible control), suggesting that this morphological trait may be associated with resistance to *C. formicarius* attack. The susceptible control (CEMSA 78-354) exhibited the shallowest tuberization depth (3.41 cm). In contrast, the genotypes V-525 (13.32 cm) and IB Morado-16 (12.50 cm) showed the highest values, which were statistically superior to the control and other intermediate genotypes. However, not all resistant genotypes followed this trend. INIVIT B-25 (4.53 cm), despite being considered resistant, did not differ significantly from the susceptible control in tuberization depth, indicating that resistance in this case may be mediated by other mechanisms (**Figure 11**).

### 3.3 Laboratory evaluation of resistance/susceptibility to *C. formicarius*

**Figure 12:**
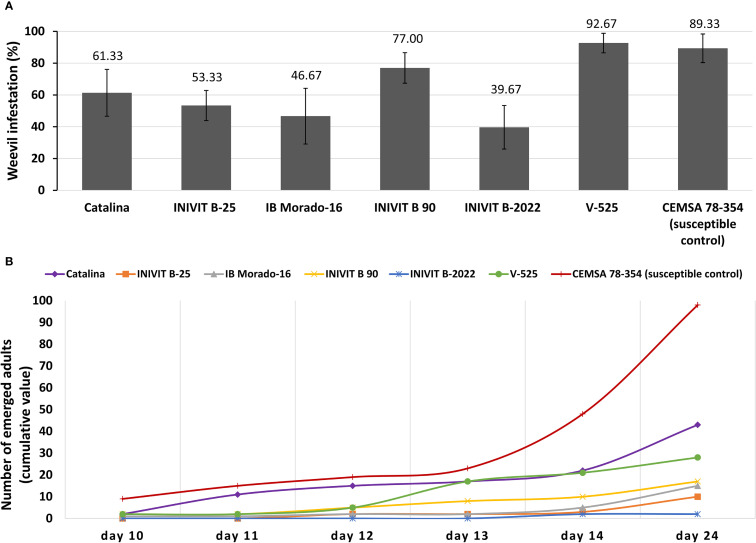
Resistance to *C. formicarius* in sweetpotato genotypes. **(A)** Infestation percentage and **(B)** Temporal dynamics of adult emergence (cumulative value).

Sweetpotato genotypes varied significantly in susceptibility to *C. formicarius* (p< 0.05). The V-525 genotype, despite being considered resistant in previous findings, showed the highest infestation percentage (92.67% ± 6.13), even surpassing the susceptible control CEMSA 78-354 (89.33% ± 8.99). This contradictory finding suggests that V-525’s resistance may be associated with mechanisms other than infestation reduction, such as tuberization depth. On the other hand, INIVIT B-2022 exhibited the lowest infestation percentage (39.67% ± 13.72), standing out as the most promising genotype for direct insect resistance. The genotypes IB Morado-16 (46.67% ± 17.52) and INIVIT B-25 (53.33% ± 9.46) showed intermediate infestation levels. Catalina (61.33% ± 14.73) and INIVIT B 90 (77% ± 9.63) occupied intermediate-high positions (**Figure 12A**).

While INIVIT B-2022 demonstrated exceptional resistance with near-total suppression of adult emergence (2.0 ± 1.5 adults across replicates), the other resistant genotypes exhibited distinct temporal patterns in weevil development. The genotypes INIVIT B-25 (10.3 ± 5.2 adults) and IB Morado-16 (15.1 ± 9.4 adults) also displayed low emergence but with different dynamics: INIVIT B-25 showed a significant delay (first adults on day 12.5 ± 7.7), while IB Morado-16 had more gradual emergence (linear increase of 1.2 ± 0.3 adults/day). This suggests their resistance mechanisms may act at different stages of the insect’s life cycle (e.g., larval toxicity vs. pupation inhibition). V-525, despite its high infestation, showed intermediate emergence (28.4 ± 12.8 adults), indicating that although the insect colonizes the tubers, its development is partially affected (p< 0.05). Catalina and INIVIT B-90 showed moderate emergence (43.2 ± 22.1 and 17.3 ± 9.3 adults, respectively), while INIVIT B-90 peaked earlier (10.2 ± 4.8 adults by day 14) (**Figure 12B**).

**Figure 13:**
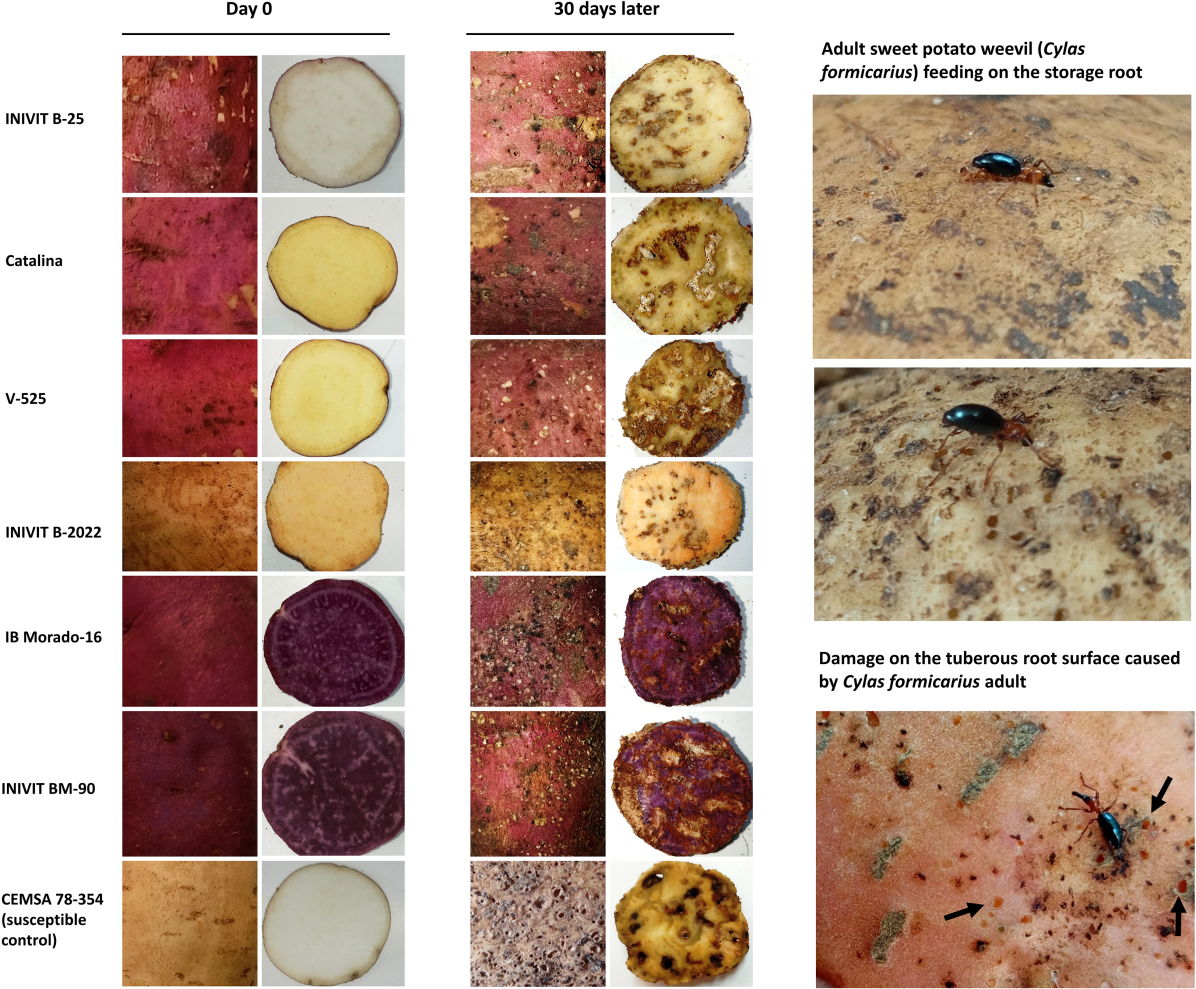
Visual Documentation of Root Phenotypic Traits and Susceptibility to *C. formicarius* Damage. Left column: Healthy roots at Day 0; Right column: Post-infestation damage at Day 30.

Visual documentation of root phenotypes (**Figure 13**) from the six most resistant genotypes reveals wide variation in flesh pigmentation (1 white, 2 yellow, 1 light orange, and 2 purple), while skin color was predominantly dark red/purple, with the exception of INIVIT B-2022 (yellow skin with orange flesh). This suggests that flesh color is not a consistent predictor of resistance. Instead, shared morphological traits such as anthocyanic skin color (5/6 genotypes), deep tuberization (5/6 genotypes), and elliptical root shape with smooth surfaces may contribute more significantly to resistance. The absence of pronounced skin defects in resistant genotypes could reduce soil surface cracking and consequently limit oviposition sites for *C. formicarius*. Notably, INIVIT B-2022 maintained its resistance (both in field and laboratory conditions), implying compensatory mechanisms.

## 4 Discussion

Morphological and morphometric characterization of the 731 sweetpotato accessions revealed broad phenotypic diversity in terms of skin and flesh coloration (**Figures 3A–D**), root and leaf shape (**Figures 4A–C**), as well as the presence of surface defects. The predominance of red and yellow skins, along with yellow, cream, and white flesh, suggests a historical preference in the selection of these traits, possibly linked to cultural aspects. The dispersion in CIELab space confirms chromatic variability, indicating a diverse genetic basis within the collection. This pattern of color distribution aligns with reports on traditional varieties from Brazil, where historical farmer selection is thought to have favored these colors due to their higher acceptance in local markets (Veasey et al., 2007).

Root morphology showed that 72.78% of the accessions exhibited surface defects such as longitudinal cracks, while 27.22% had smooth surfaces (**Figure 4B**). On the other hand, leaf morphometry revealed notable variability in terms of circularity, aspect ratio, and leaf contour complexity. Results from South Carolina on 731 sweetpotato accessions also showed broad phenotypic variability in leaves, with 47.3% classified as lobed and 32.3% as cordate (Jackson et al., 2012).

The results demonstrated that resistance to *C. formicarius* is not strongly correlated with specific morphological traits (**Figure 8C**), such as skin or flesh color, leaf shape, foliar pigmentation, or flowering. However, interesting patterns were observed: resistant accessions (6.5%) tended to have lighter skins and less flesh pigmentation (though this trend did not persist in later stages), as well as greater root firmness. The presence of surface defects showed a moderate correlation with susceptibility (**Figure 9D**), suggesting that roots with pronounced defects may facilitate crack formation in the soil and insect access. This aligns with findings by Waddill and Conover (1978) and Talekar (1987), who reported instability in sweetpotato genotype resistance due to environmental influences and the species genetic complexity. The predominance of red and yellow skins in resistant accessions could be related to the accumulation of secondary compounds, such as anthocyanins, which have been associated with defense mechanisms in other crops (Stevenson et al., 2009).”

Field evaluations identified six resistant genotypes (**Figure 10B**), with INIVIT B-25 and INIVIT B-2022 standing out for combining high yields with low infestation percentages. Tuberization depth (>10 cm) emerged as an escape mechanism in five of the six resistant genotypes (**Figure 11**), as deeper roots are less exposed to the insect by avoiding soil cracking during tuber formation. However, the case of INIVIT B-25 which exhibited shallow tuberization yet significant resistance suggests the involvement of other factors, such as biochemical mechanisms or antixenosis.

A recent study found that high-yielding genotypes may be more susceptible to weevil infestation due to greater root exposure (Osaru et al., 2023). Although this observation was very common in our study (**Figures 10A, B**), there are exceptions where high yield and resistance were combined in the same genotype. Studies support our findings, including research from Nigeria (Leuschner, 1980), United States (Mullen et al., 1981) and Tanzania (Kagimbo et al., 2020). Studies in Puerto Rico correlated elongated tuber shapes and greater root depth with lower infestation. Elongated root varieties showed 41% damage compared to 52% in round tuber genotypes (Cabrera et al., 1990). Additionally, key studies in Taiwan demonstrated that none of the 1,000 evaluated accessions exhibited consistent weevil resistance across multiple seasons or locations. Some initially promising accessions later proved susceptible in follow up trials, suggesting that resistance may not exist in traditional sweetpotato germplasm (Talekar, 1987).

Controlled environment trials revealed marked differences in genotype responses to weevil attack. Controlled infestation assays identified three primary defense mechanisms against *Cylas formicarius*, antixenosis (non-preference), antibiosis (adverse effects on the insect), and physical escape. For INIVIT B-2022, results showed a 60.3% reduction in infestation and emergence of only 2 adults (**Figure 12B**), indicating simultaneous antixenosis and antibiosis. In contrast, V-525 though resistant in the field was highly susceptible in the laboratory (**Figure 12A**), suggesting its resistance may depend on ecological factors like tuberization depth. Temporal dynamics of adult emergence also varied among genotypes, with INIVIT B-25 delaying larval development (**Figure 12B**), pointing to distinct modes of resistance. These results reinforce the multifaceted nature of plant-insect interactions, which may involve both morphological traits and biochemical defenses. Furthermore, CEMSA 78-354 (susceptible control) confirmed the absence of both mechanisms, with high infestation (89.33%) and adult emergence (98 individuals) (**Figures 12A, B**).

Our results align with studies reporting significant differences in susceptibility to *C. formicarius*, where adult emergence ranged from 8 to 230 per genotype (Waddill and Conover, 1978). Laboratory tests have also documented genotypes reducing adult emergence by up to 78% and delaying development by 4–6 days (Leuschner, 1980). Under laboratory conditions (**Figure 2**), tuberous roots are exposed to forced and constant infestation pressure, which does not reflect the insect’s natural selectivity in the field.

Similarly, the genotype IB Morado-16 exhibited moderate infestation (46.67%) but low adult emergence (15 individuals) (**Figure 12**). This indicates that while the insect colonizes the tubers (low antixenosis, failing to prevent oviposition), some degree of antibiosis (delayed development) occurs, possibly due to toxins inhibiting pupation or digestive enzymes affecting larvae. This resistance profile is notably similar to that described for the Ugandan variety ‘New Kawogo,’ where larvae developed on this variety showed higher mortality and a prolonged life cycle, suggesting a toxic effect associated with its tissues (Stevenson et al., 2009). Further, they confirmed that high concentrations of caffeic and coumaric acid esters (hexadecyl caffeate and hexadecyl coumarate) in the latex of ‘New Kawogo’ (43 mg/ml and 42 mg/ml, respectively) are key compounds in resistance (Stevenson et al., 2009).

A laboratory study in Indonesia revealed that 66.7–100% of roots exhibited surface damage, while internal damage intensity ranged from 14.0 to 76.6%, classifying some genotypes as moderately resistant. Additionally, they suggested that weevil oviposition preference is directly linked to the damage caused (Mau et al., 2021).

A recent study in Uganda evaluating 30 sweetpotato genotypes found that root damage severity had a significant negative correlation with root stalk length (r = −0.6), identifying this trait as the most reliable morphological predictor of sweetpotato weevil resistance (Osaru et al., 2023). Our results confirm this observation, as deep tuberizing varieties generally possess long stalks and vice versa (**Figure 11**). However, while this is a physical escape mechanism, resistance does not depend solely on a single morphological trait. Weevil resistance is highly influenced by environmental, physiological, and genetic factors, making it unstable under varying conditions (e.g., drought), and the weevil may circumvent this physical barrier. Thus, we propose that identifying varieties with higher resistance requires combining multiple traits in the same genotype:

1. Root stalk length or tuberization depth (>10 cm).
2. Smooth, defect-free skin.
3. Preferably elliptical tuber shape (not round).
4. Concurrent antixenosis and antibiosis mechanisms (e.g., INIVIT B-2022).

Given the current absence of globally available varieties with true resistance, it is recommended using genotypes that combine these traits.

## 5 Conclusion

This study demonstrates that resistance to *C. formicarius* in sweetpotatoes is a complex phenomenon influenced by multiple factors, including morphological traits, escape mechanisms, and potential biochemical defenses. The identification of genotypes like INIVIT B-2022 and INIVIT B-25 with contrasting yet effective resistance profiles provides valuable opportunities for developing resilient varieties. Future research should integrate molecular and metabolic approaches to unravel the genetic basis of these mechanisms, particularly focusing on the biochemical properties of INIVIT B-25’s shallow tuberization resistance and INIVIT B-2022’s dual antixenosis-antibiosis effects. These findings offer practical solutions for Caribbean farmers while advancing global understanding of sweetpotato-weevil interactions. Future research should integrate molecular and metabolic approaches to unravel the genetic basis of these mechanisms.

## Data Availability

The raw data supporting the conclusions of this article will be made available by the authors, without undue reservation.
